# 

**Published:** 1915-01

**Authors:** 


### CONTENTS.

ORIGINAL COMMUNICATIONS—

Address of the Retiring President of the Fulton County Medical Society, By Stephen D. Barnett, M. D. .......................... 445

Milk Therapy in Nutritional Disturbances, by Howard Bucknell, M. D. .......................... 451

Physical Exercises in the Treatment of Nervous Diseases, by Theodore Toepel, M. D. .............. 457

Meeting of the Fulton County Medical Society, by R. R. Daly, M. D. .......................... 462

EDITORIAL .......................... 469

NEWS AND NOTES .......................... 470

SELECTIONS AND ABSTRACTS .................. 480

